# Supplementary material for: Association between oral microbiome and seven types of cancers in East Asian population: a two-sample Mendelian randomization analysis
Source: Front Mol Biosci. 2023 Nov 21;10:1327893. doi: 10.3389/fmolb.2023.1327893 (PMC10702768; doi:10.3389/fmolb.2023.1327893)
Supplement: Supplementary file 1 [file DataSheet1.ZIP › Supplementary Materials/MR plots of saliva/saliva═╝/ovarian cancer/pheno.3313_to_ovarian cancer_forest.pdf]

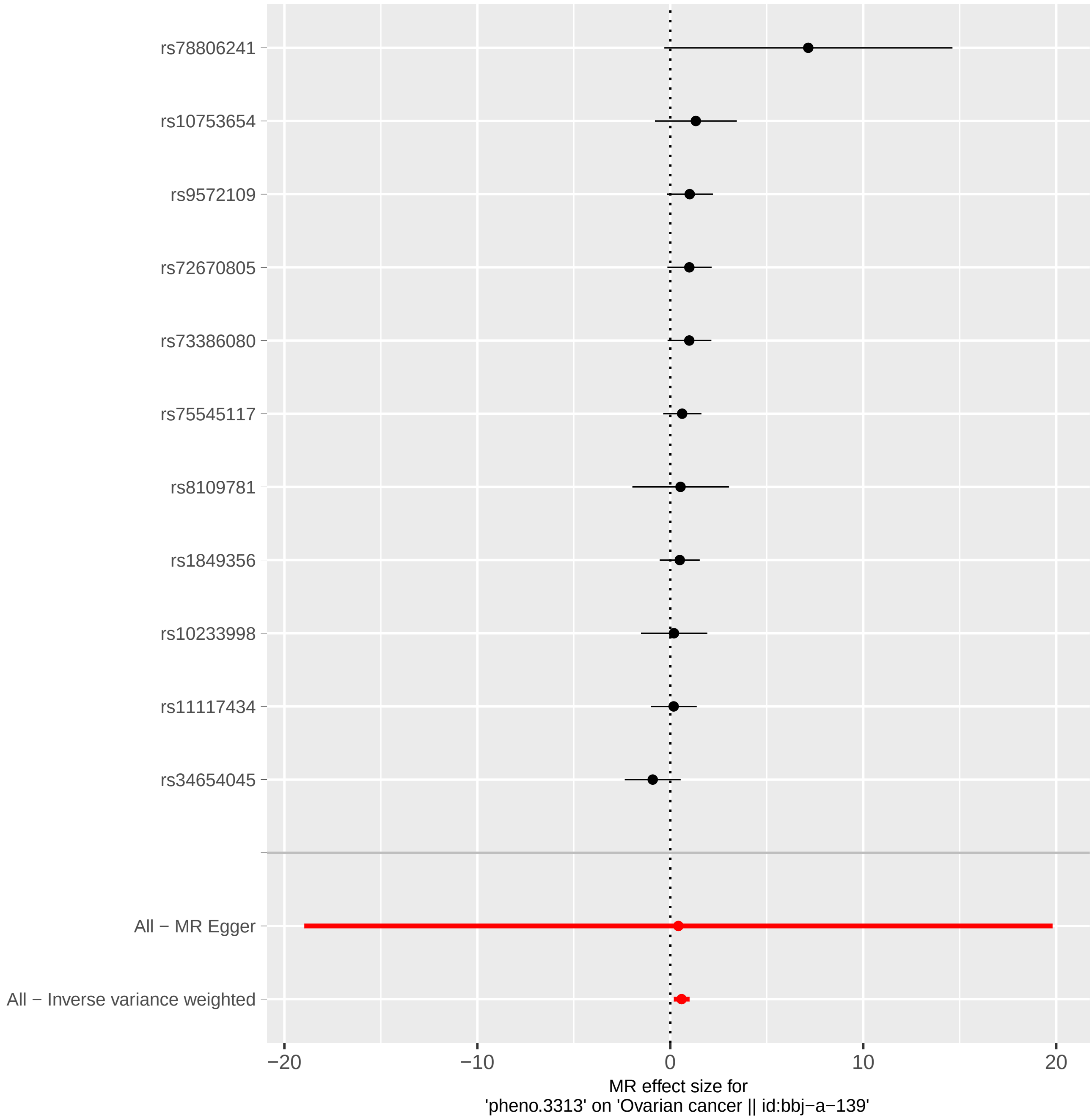
rs78806241
rs10753654
rs9572109
rs72670805
rs73386080
rs75545117
rs8109781
rs1849356
rs10233998
rs11117434
rs34654045
All − MR Egger
All − Inverse variance weighted
−20
−10
0
10
20
MR effect size for
'pheno.3313' on 'Ovarian cancer || id:bbj−a−139'
